# Rightward-biased hemodynamic response of the parahippocampal system during virtual navigation

**DOI:** 10.1038/srep09063

**Published:** 2015-03-12

**Authors:** Travis E. Baker, Akina Umemoto, Adam Krawitz, Clay B. Holroyd

**Affiliations:** 1 Department of Psychiatry University of Montreal Quebec, Canada; 2 Department of Psychology University of Victoria British Columbia, Canada

## Abstract

Phase reset of parahippocampal electrophysiological oscillations in the theta frequency range is said to contribute to item encoding and retrieval during spatial navigation. Although well-studied in non-human animals, this mechanism is poorly understood in humans. Previously we found that feedback stimuli presented in a virtual maze environment elicited a burst of theta power over right-posterior areas of the human scalp, and that the power and phase angle of these oscillations were greater following right turns compared to left turns in the maze. Here we investigated the source of this effect with functional magnetic resonance imaging. Consistent with our predictions, we found that 1) feedback encountered in the maze task activated right parahippocampal cortex (PHC), 2) right PHC was more activated by rewards following right turns compared to left turns in the maze, and 3) the rightward-biased activation was more pronounced in individuals who displayed good spatial abilities. These findings support our previous electrophysiological findings and highlight, in humans, a role for PHC theta oscillations in encoding salient information for the purpose of spatial navigation.

When animals navigate a spatial environment, theta oscillations produced by the parahippocampal cortex (PHC) contribute to the formation of spatial memories^1^,^2^. In particular, the phase of firing of PHC neurons relative to locally recorded oscillations in the theta frequency range (5–12 Hz in animals) of the local field potential are thought to constitute a temporal mechanism by which spatial information is encoded for the purpose of spatial navigation^3^. It has been suggested that this mechanism facilitates encoding and retrieval by enabling spatially relevant memories to be encoded with respect to different phases of the theta rhythm^4^. For example, the phase of the theta rhythm is reset by the occurrence of salient events or cues such as rewards and navigationally-relevant landmarks, which facilitates the encoding of this information with respect to the peaks and troughs of the theta oscillations^5^ via enhanced long-term potentiation^6^. Yet although this mechanism is relatively well understood in non-human animals, little is known about it in humans.

Recently, we demonstrated that the presentation of reward-related feedback stimuli in a virtual environment induces a burst of EEG oscillations in the theta frequency range over posterior areas of the human scalp^7^. Strikingly, we found that feedback stimuli encountered following right turns in the maze, when compared to feedback stimuli encountered following left turns in the maze, elicited a stronger theta response with a relatively larger phase angle of about 13–15° degrees. Further, source localization of this effect pointed to a generator in the right PHC. These differences in the power and phase of theta oscillations were unrelated to the retinotopic position of the feedback (which always appeared at fixation) and to the motor activity of the responding hand (as demonstrated by a control condition that did not produce the effect^7^), but rather reflected the spatial position of the feedback relative to the participant's navigational trajectory within an egocentric frame of reference. Further, participants who successfully drew the spatial layout of a complex maze from memory produced a larger effect compared to participants who could not reproduce the maze, suggesting that the effect is sensitive to individual differences in the ability to represent spatial relationships^8^. Taken together, these findings support the proposal that the theta effect is produced by a neural system for spatial navigation and reflects individual differences in the efficacy of this system. Based on the non-human animal research described above, we proposed that reward-related stimuli in the maze induced a partial phase reset (i.e. concomitant increases in phase coherence and spectral power relative to baseline) of the PHC theta rhythm with relatively greater power and phase angle following right turns compared to left turns in the maze, which we term here the *theta effect*. This phenomenon is also observed in the time domain as a component of the event-related brain potential (ERP), the topographical N170^7^,^8^.

However, this interpretation is complicated by the inverse problem − that any given voltage distribution across the scalp can be accounted for by an infinite number of possible configurations of intracranial sources^9^,^10^. Thus, although our previous source localization findings implicate right PHC in producing the theta effect, this inference remains to be confirmed. Here we addressed this issue with fMRI. Specifically, we reasoned that converging evidence across human fMRI and human electrophysiological studies would provide relatively solid evidence that the theta effect is in fact generated in the right PHC. Participants engaged in a modified version of the virtual T-maze task^7^,^8^ previously used to demonstrate the theta effect (Figure 1). In the “T-maze condition”, participants were asked to find rewards by selecting between two alley options in the virtual maze. In the “No-maze condition”, the participants engaged in a task that was formally identical to the T-maze task except that the imperative and feedback stimuli were displayed against scrambled images of the maze environment, which dissociated these events from their spatial context. Individual differences in spatial ability were assessed prior to scanning using a virtual environment test battery and a questionnaire that assessed everyday spatial abilities.

On the basis of our previous findings we predicted the following. If the theta effect were in fact produced by a PHC system for spatial navigation in the right hemisphere, we predicted that, first, feedback stimuli would produce a greater hemodynamic response in the right PHC when presented in the T-maze condition compared to the No-maze condition, and second, that the feedback stimuli would produce a greater hemodynamic response in right PHC when following right turns compared to left turns in the maze. Further, we predicted that if the strength of the theta effect in fact reflects individual differences in spatial abilities mediated by the PHC, then feedback stimuli would elicit stronger hemodynamic responses for individuals with good spatial abilities compared to individuals with poor spatial abilities (please see SOM for behavioural results).

## Results

### Prediction 1

First, we predicted that if the theta effect is produced by a PHC system for spatial navigation in the right hemisphere, then feedback stimuli would elicit a greater hemodynamic response in the right PHC when presented in the virtual environment (T-maze condition) as compared to being presented against scrambled images of that same environment (No-maze condition). A whole-brain contrast between T-maze and No-maze trials time-locked to feedback onset confirmed this prediction: a contrast of the hemodynamic response to feedback stimuli presented in the virtual T-maze task compared to feedback stimuli presented in the No-maze task revealed statistically significant activations in the right PHC (t_(15)_ = 8.31, p < .05 FWE-corrected; voxel size: 20) (Figure 2, Table 1). Statistically significant activations were also observed in left PHC, bilaterally in the middle temporal-occipital cortex, and in the right precuneus (Figure 2, Table 1), regions commonly activated during virtual navigation^11^,^12^,^13^. By contrast, middle-occipital cortex and the right cuneus were more activated in the No-maze condition than in the T-maze condition (Table 1). Consistent with previous reports, these results demonstrate that the same virtual environment utilized in our previous studies, which we argued elicited a partial phase reset of theta oscillations in right PHC, do in fact activate right PHC.

### Prediction 2

We recently demonstrated that reward-related stimuli encountered in a virtual maze elicited relatively greater power in the theta frequency range, with a relatively greater phase angle, when these feedback stimuli were found following right turns compared to left turns in the maze (the theta effect^7^). On the premise that the enhanced theta power reflected an intensified neural response to the eliciting stimuli, here we tested the prediction that the right PHC would show a stronger hemodynamic response to feedback processing in the right alley compared to the left alley of the T-maze. As would be expected given the motor demands of the task, a whole-brain analysis revealed activation in the left primary motor cortex to feedback encountered in the right alley (t_(15)_ = 8.38, p < .05 FWE-corrected; voxel size: 19), and activation in the right motor cortex to feedback encountered in the left alley (t_(15)_ = 9.94, p < .05 FWE-corrected; voxel size: 126). No other brain areas showed a statistically-significant difference in activation to feedback presented in the left and right alleys in the T-maze. To test the prediction specifically, we conducted a ROI analysis focussed on the right PHC. The analysis confirmed that a region within the posterior right PHC (t_(15)_ = 5.25, p < .05 FWE-corrected; voxel size: 3) was relatively more activated by feedback stimuli presented in the right alley compared to the left alley of the T-maze. Further, for the purpose of comparison, the opposite contrast revealed no voxel values that were stronger for feedback stimuli presented in the left alley compared to the right alley of the maze. Additional analyses conducted separately on ROIs focused on the left PHC and on the other activated regions observed in the T-maze vs No-maze contrast (right precuneus and bilateral middle temporal cortex) also did not reveal any statistically significant activations for either the left alley-right alley or right alley-left alley contrasts. These findings indicate that the right PHC was more activated by feedback stimuli presented following rightward compared to leftward turns in the virtual environment, consistent with our predictions.

We further explored these findings by relaxing the threshold (p < .005, uncorrected) to examine whether or not activity in the right PHC was confined to the posterior region of the PHC. This exploratory analysis yielded two distinct clusters within the PHC, a posterior PHC cluster (t_(15)_ = 5.25, p < 0.005, uncorrected; voxel size: 37), and an anterior PHC cluster (t_(15)_ = 3.28, p < 0.005, uncorrected; voxel size: 9) (Figure 3). As a check, additional analysis revealed that no region of right PHC was more activated for feedback stimuli encountered in the left alley compared to the right alley of the T-maze, and that the cortical regions activated by the T-maze vs No-Maze contrast also did not display any significant voxels at this threshold for both the left alley-right alley and right alley-left alley contrasts. These results confirm our central prediction that right PHC would be more activated by feedback stimuli encountered in the right arm of the virtual maze compared to the left arm of the virtual maze. Interestingly, the anterior and posterior PHC clusters related to spatial navigation were distinct from the PHC cluster identified by the T-maze vs No-maze contrast, suggesting that different regions in the PHC may mediate different functions associated with spatial navigation.

### Prediction 3

In the previous study, we demonstrated that the theta effect is sensitive to individual differences in spatial navigation (when measured in the time-domain as the topographical N170 latency effect^7^). In particular, we found a reduced theta effect for participants who were impaired at reconstructing a complex maze from memory. Here we predicted that the hemodynamic response of right PHC to feedback stimuli would likewise reflect individual differences in spatial ability. Prior to the fMRI session, participants were asked to engage in a virtual complex maze task and then reconstruct the complex maze from memory^7^. We found that after the first block of 25 trials, 8 participants (4 males) were able to reconstruct the maze without making any major drawing errors (Drawers: error score = 0), whereas 8 participants (4 males) could not reconstruct the maze (Non-drawer: error score = 3.4, max score = 4) (Figure 4). Questionnaire responses indicated that Non-drawers not only performed worse on the spatial navigation tasks, but were also poor navigators in general, suggesting that spatial navigation was impaired in these individuals in both laboratory and natural settings, consistent with previous observations^7^. A three-factors repeated-measures ANOVA on the hemodynamic response with the PHC region (posterior, anterior) and feedback location (feedback left alley, feedback right alley) as within-subject factors, and spatial group (Drawer vs. Non-Drawer) as between subject factors (with Age and Sex included as covariates) revealed a main effect of feedback location (F_1, 12_ = 9.95, p < .01), an interaction between feedback-location and spatial group (F_1, 12_ = 4.71, p < .05) and an interaction between PHC region and feedback location (F_1, 12_ = 5.31, p < .05) (Figure 5). The other main effects and interactions were not significant (p > .05).

Paired *t*-tests on the event-related average data confirmed that the right PHC was significantly more activated when feedback was presented in the right alley (M = .06, SE = .02) compared to the left alley (M = .02, SE = .02) (p < .005, *bonferroni-corrected*). Further, the Drawer group on average exhibited a stronger PHC activation when feedback was presented in the right alley (M = .12, SE = .04) compared to when feedback was presented in the left alley, (M = .06, SE = .03) (p < .05, *bonferroni-corrected*), but this difference was not observed for the Non-Drawer group (right alley, M = .02, SE = .03; left alley M = .02, SE = .03) (p > .1, *bonferroni-corrected*). Finally, although the ANOVA revealed a PHC cluster x feedback-location interaction, the results of a post-hoc analysis on the percent signal change difference in PHC activation for feedback found in the left versus right alleys was not statistically different for the posterior and anterior PHC clusters (p > .1, *bonferroni-corrected).*

Exploratory analyses also revealed that posterior PHC activity was positively correlated with performance on the path integration task (r = .613, p = .02), and that anterior PHC activity was negatively correlated with performance on the Cognitive Map formation task (r = −.591, p < .05; note that lower scores on Cognitive Map test indicate better performance), while both posterior (r = .546, p = .02) and anterior (r = .543, p = .02) regions were positively correlated with everyday spatial ability. Notably, no other associations were observed between the PHC clusters and other measures of spatial ability, object recognition, face recognition, and facial expression recognition (p > .1).

## Discussion

A large body of evidence from rodent studies indicates that PHC updates and stores contextual information related to spatial navigation according to the phase of local electrophysiological oscillations in the theta frequency range^4^. In line with these observations, neural network models of parahippocampal function depend on a theta phase-reset mechanism to correct for the cumulative error associated with path integration^14^. Other computational models depend on theta phase reset to simulate context-sensitivity of neurons that fire in response to salient environmental events such as stopping, reward and turning locations^5^. Further, in line with previous suggestions that this brain area in humans constitutes the “Parahippocampal Place Area”^15^ or the “Parahippocampal Spatial Scene Area”^16^, recent primate intracranial^17^,^18^,^19^ and human fMRI^20^ studies are also suggestive of theta phase coding. For instance, increases in PHC activity were observed while human participants learned to locate rewards in a virtual maze^21^, implicating a role for this brain area in representing and retaining egocentric spatial information^22^. In another noteworthy study that used a virtual environment modeled after a rat foraging task, Doeller et al. (2010) observed that the fMRI BOLD response in human right PHC exhibited a speed-modulated six-fold rotational symmetry in running direction as predicted by theoretical models of theta phase coding. Variations in the structure and function of PHC and surrounding structures have also been observed to contribute to individual differences in human spatial navigation^23^.

In line with these observations, we recently demonstrated that the presentation of reward-related stimuli in a virtual environment induces a burst of EEG oscillations in the theta frequency range over posterior areas of the human scalp, and that the power and phase angle of the theta rhythm is significantly larger when participants receive feedback following right turns in the maze compared to left turns in the maze (the theta effect). Source localization of this effect pointed to a generator in the right PHC^7^. This finding resonates with the previous observations, namely, that the right PHC contributes to topographical learning in humans^22^, feedback and emotion-modulated spatial learning^24^ and memory^25^, and that these functions depend on the temporal firing of PHC neurons in relation to the phase of the theta rhythm^4^.Understood in this context, the theta effect reflects a phase-coded signal by PHC for encoding contextual information about reward location in the environment. The present fMRI experiment was intended to provide converging evidence for this proposal.

We tested three predictions that follow from our hypothesis. First, we predicted that right PHC would respond more strongly to feedback stimuli encountered in a virtual maze as compared to the same stimuli presented in the absence of any spatial context. Consistent with this prediction, we found that the right PHC was more activated by feedback stimuli encountered in the same virtual T-maze that we utilized in our previous EEG studies, when compared to feedback stimuli presented in the No-maze task. This result indicates that the task previously utilized to produce the theta effect, the source of which was localized to the right PHC^7^, does in fact activate the right PHC. Second, we predicted that the right PHC would generate a stronger hemodynamic response to feedback stimuli encountered in the right alley compared to the left alley of the T-maze^7^. This prediction was confirmed. When we explored this phenomenon further by reducing the statistical threshold, we found that the contrast also activated an anterior region in the right PHC. This difference was not observed in left PHC nor in any of the other brain regions that were also sensitive to the maze vs. no-maze contrast. Neither were any of these regions more activated by rewards presented in the left alley of the maze compared to the right alley of the maze. Our findings were thus specifically consistent with the theta effect and provide converging support for our previous source localization analysis, which identified right PHC as the source of the effect.

Third, as predicted, we observed that individuals with good spatial ability relative to individuals with poor spatial ability (as assessed by performance on a complex maze reconstruction task) produced a stronger hemodynamic response to rewards found in the right arm of the maze compared to the left arm of the maze. These differences in brain activation also mirrored differences in every-day spatial abilities such as finding one's way in the environment and learning the layout of a building or city^26^, suggesting that individual differences in PHC function span laboratory and non-laboratory settings. Complementing these findings, we found that posterior PHC activity was associated with performance on a path integration task, that anterior PHC activity was associated with performance on a Cognitive Map formation task, and that both regions were associated with everyday spatial ability. These observations are consistent with previous reports that individual differences in spatial ability reflect natural variability in PHC function^23^,^26^,^27^,^28^, and support and extend our previous finding that participants who successfully recalled the layout of a complex virtual maze from memory produced an enhanced theta effect (Experiment 3 in Ref. 7).

Taken together, this evidence suggests that a stimulus-induced increase in power and phase consistency of theta oscillations contributes to the differential response by right PHC to right vs. left feedback locations in the maze, an inference that dovetails with previous conceptions about the role of the theta reset function in the PHC with respect to spatial navigation^4^. It is interesting to note that reducing the statistical threshold revealed 3 distinct regions within the right PHC: the T-maze versus No-maze contrast revealed a relatively large cluster of activation in a central region of PHC, whereas the right alley vs. left alley contrast revealed clusters of activation in posterior and anterior PHC. In contrast to the view that the PHC is a single coterminous region^29^ that performs a unitary spatial function^30^, our findings support the idea that the PHC is composed of multiple functional components along its posterior-anterior axis^30^,^31^,^32^. For example, the Parahippocampal Place Area^15^ (the posterior portion of the PHC that extends into the lingual gyrus) responds preferentially to pictures of places^31^, whereas the entorhinal cortex (the anterior portion of the PHC) has been shown to play a more general role in spatial memory encoding^32^. And a recent functional connectivity study reported evidence that anterior and posterior PHC exhibit distinct connectivity patterns, with the posterior PHC more strongly coupled to activity in occipital lobe visual regions, and the anterior PHC more strongly coupled to activity in parietal lobe regions implicated in spatial processing^30^. In the context of these observations, we speculate that the clusters of activation identified in the present study may reflect these diverse PHC regions, the Parahippocampal Place Area (posterior PHC cluster) and entorhinal cortex (anterior PHC cluster) respectively, and that enhanced synchronization of theta activity may play a role in regulating information flow within the PHC circuitry in a way that optimizes event encoding.

Our results confirmed our prediction that right PHC would be more activated by feedback found following rightward turns compared to leftward turns in a spatial environment, but the posterior PHC activation was relatively small (3 voxels, FWE-corrected), and the anterior PHC activation was observed only at the higher threshold (p < .005, uncorrected). These weak activations are not surprising given that in our previous studies the differences in theta power and phase angle between reward locations were also small, albeit consistently observed across multiple experiments. Further, when we expanded our analysis to include other brain areas sensitive to spatial processing, none of them exhibited greater activation to feedback following right vs. left turns, nor greater activation to feedback following left vs. right turns, even at the lower threshold. PHC activation was also not correlated with individual differences in face and object processing. These observations speak to the statistical robustness of the result.

A challenging question is why the PHC in fact responds differently when salient information is encountered following right vs. left turns in a virtual environment. Why do right turns in the T-maze induce a relatively larger increase in theta power and phase angle, together with an increased PHC BOLD response, to feedback stimuli that are subsequently encountered in that arm of the maze? Presently we can only speculate about the answer to this riddle. Nevertheless, given that humans and non-human animals have been widely observed to exhibit a navigational turning bias^33^,^34^, we suspect that the PHC system may bias rightward trajectories by encoding phase angle with respect to a rightward-anchored reference point^8^. We hope that the results of this study will motivate future research on this issue.

## Conclusion

Substantial evidence indicates that the theta rhythm plays a vital role in PHC information processing and memory formation. Theta phase reset likely contributes to this process but the importance of the reset mechanism for memory formation in humans has yet to be established. Our study provides converging evidence for the proposal that theta phase coding and resetting contribute to human PHC activity during spatial navigation. These findings hold out promise for integrating experimental, computational, and theoretical analyses of animal PHC function together with human cognitive neuroscience research, as well as with research on memory-related disorders such as Alzheimer disease and topographical agnosia.

## Methods

### Participants

Sixteen undergraduate participants (8 male, 8 female; aged 18–26, M = 19.8, SD = 2.6; 14 right-handed) were recruited from the University of Victoria and each received course credit as well as a monetary bonus associated with the experimental task. The amount of money depended on the probability of the feedback, as described below. All participants had normal or corrected-to-normal vision, none reported a history of head injury, and all reported experience playing first-person perspective video games. The research was approved by the joint University of Victoria and the Royal Jubilee Hospital ethics committee in accordance with the Declaration of Helsinki. All participants gave written informed consent and underwent pre-scan screening.

### fMRI Task

**Figure 1:**
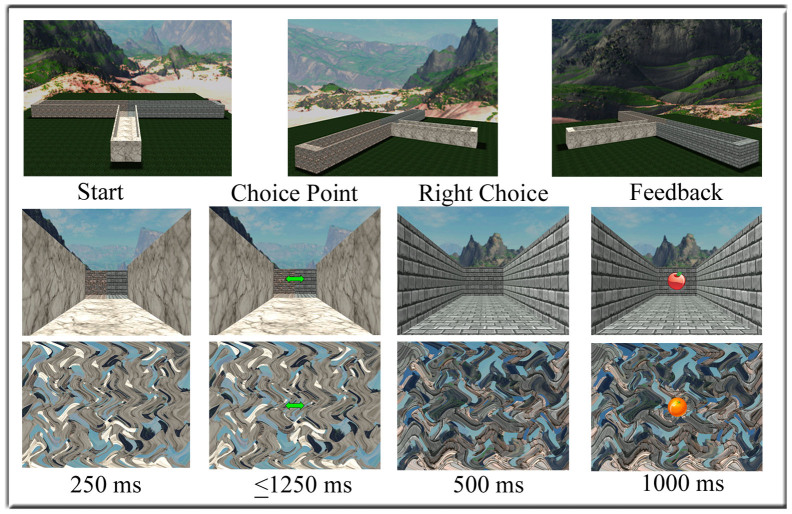
Top: view of T-maze from above. Bottom: sequence of events comprising an example trial of the T-maze (middle panel) and No-maze (bottom) tasks. Top line indicates stimulus type and the bottom line indicates stimulus duration; the double arrow remained visible until the button press.

**Figure 2:**
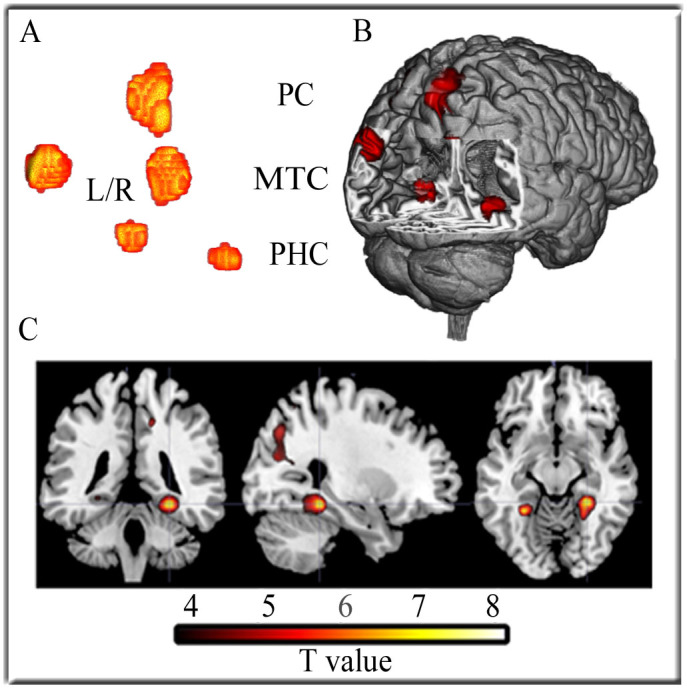
Results of the whole brain analysis for the Maze vs. No-maze contrast. Regions that are more active for the Maze condition compared to the No-maze condition (A) are superimposed on a T1-weighted anatomical 3D image (B) using MRIcrogl software. (C) 2D images are shown aligned to the voxel of peak activation in right parahipocampal cortex for coronal (left), sagittal (middle), and horizontal (right) slices. The statistical threshold is set at p < .05 (FWE-corrected). PC = Precuneus, MTC = Medial Temporal Cortex, and PHC = Parahippocampal Cortex.

**Figure 3:**
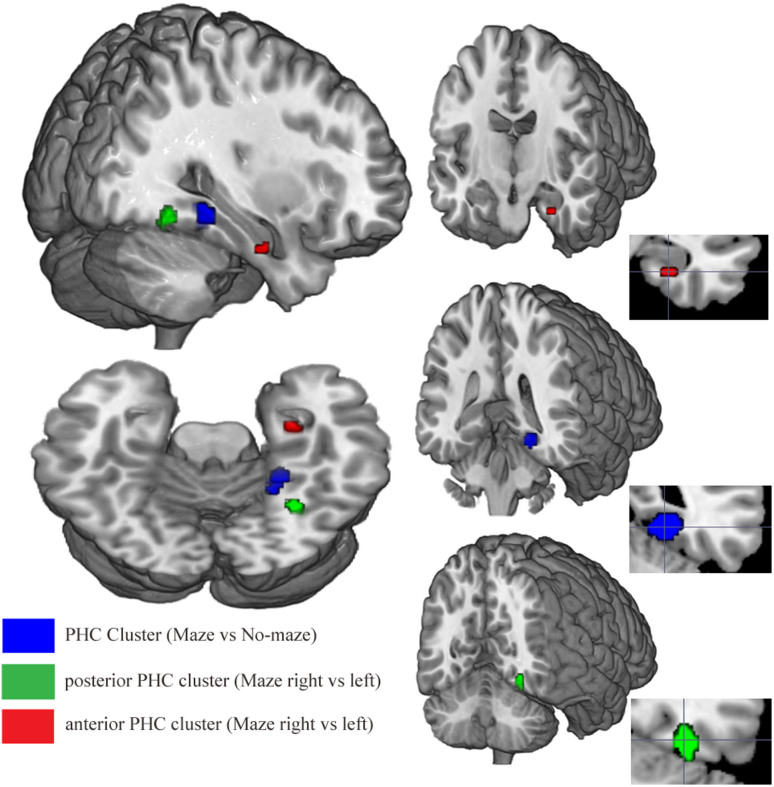
PHC clusters identified by the *Maze vs. No-maze* (blue) and *Maze right vs. Maze* *left* (green-posterior, red-anterior) contrasts are superimposed on a T1-weighted anatomical 3D image using MRIcrogl software. For the purpose of illustration, a ROI mask was created for each PHC cluster using a statistical threshold of p < .005 (uncorrected) and displayed accordingly. Coronal images are aligned according to the most statistically significant voxel for each cluster.

**Figure 4:**
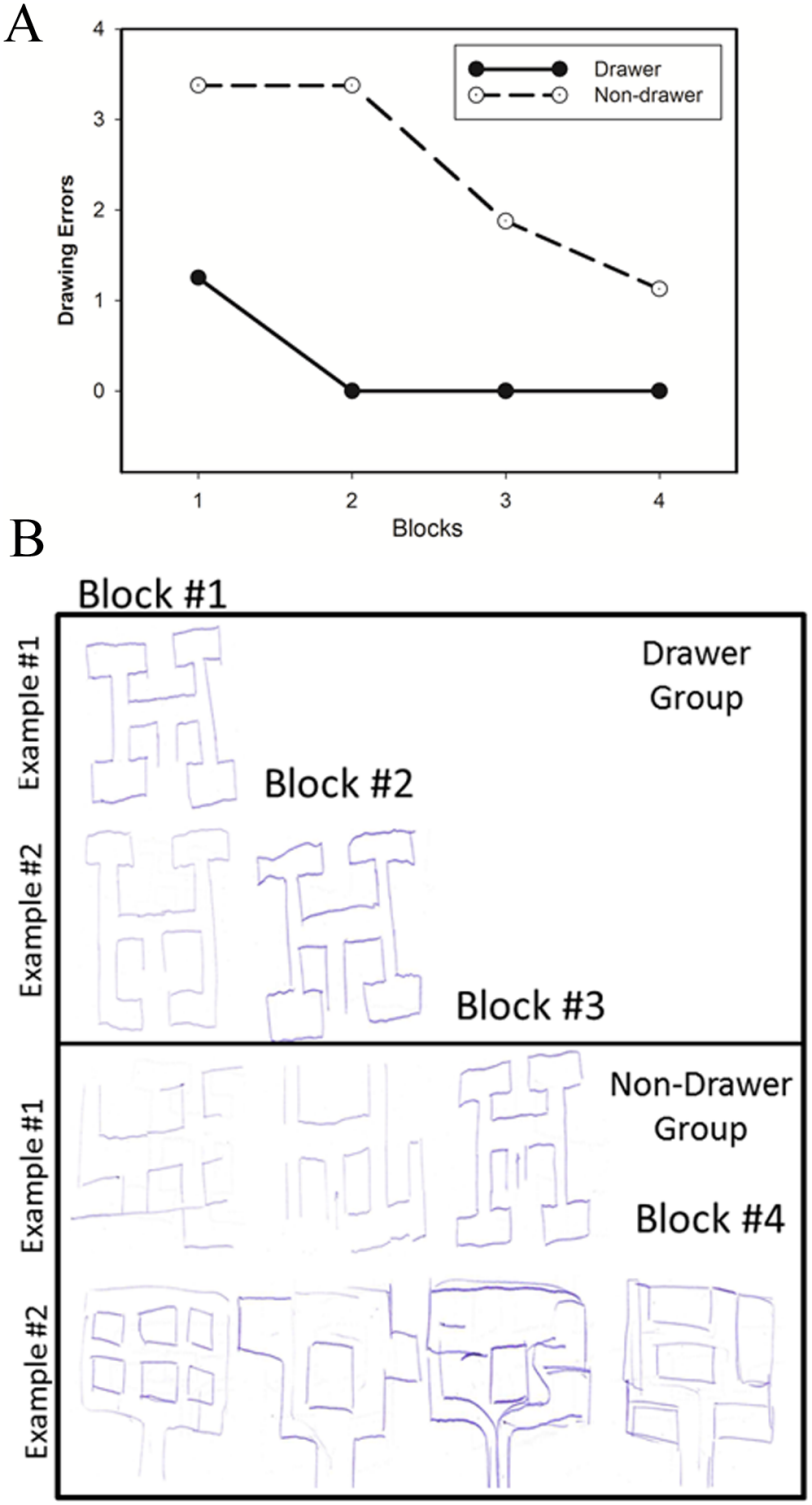
Reconstruction Task results. (A) Number of errors committed for each block of trials for participants who were classified as either Drawer or Non-drawer. (B, Top panel) Two reconstruction examples of the Drawer group after 25 trials (Block 1) and after 50 trials (Block 2). (B, Bottom panel) Two reconstruction examples of the Non-Drawer group after 25 trials (Block 1), 50 trials (Block 2), 75 trials (Block 3) and 100 trials (Block 4).

**Figure 5:**
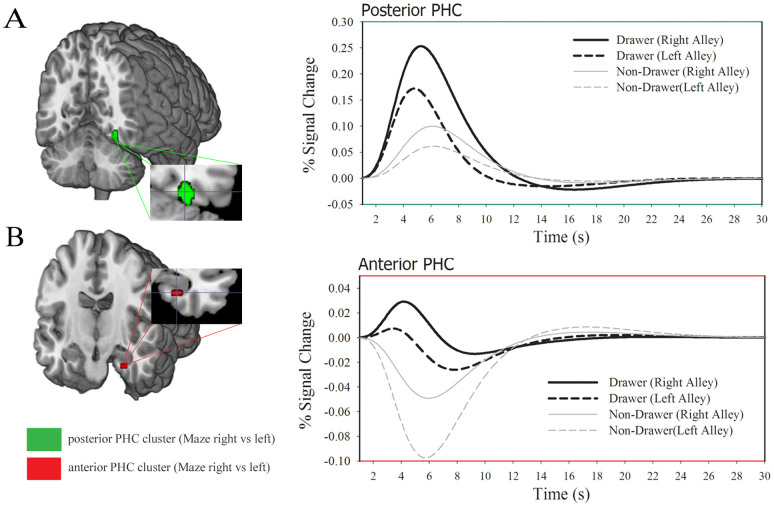
The results of the ROI analysis associated with posterior (green) and anterior (red) parahippocampal cortex (PHC) for the contrast between feedback found in the right vs. left arm of the maze. Event-related averages extracted from posterior PHC (A) and anterior PHC (B) for the Maze right vs. Maze left contrast. Black solid (right alley) and dashed (left alley) time courses for the Drawer group, and grey solid (right alley) and dashed (left alley) for the Non-Drawer group, respectively.

In the “T-maze condition”, participants were first presented with the stem image, followed by a green double arrow appearing in the center of the stem image at the maze intersect (Figure 1, bottom). Participants were instructed to press button 1 with their left index finger to select the left alley or to press button 2 with their right index finger to select the right alley. Following their response, the image of the selected alley appeared for a duration of 500 ms. Then, an apple image or an orange image appeared in the center of the alley image (1.6° wide and high). Together, the alley and fruit image remained on the screen for 1000 ms. Note that the feedback stimuli indicated whether the participants received a financial reward (5 cents) or nothing (0 cents), and that, unbeknownst to the participants, the type of feedback was selected at random (50% probability) and was counterbalanced across participants. Further, the stem and the alleys in the images were composed of different types of brick, the texture and color of which were held constant for the stem but which were counterbalanced across subjects for the left and right alleys. Background images of mountain views were also counterbalanced across participants, preventing against a differential brain response across subjects to the surface details of the left vs. right arms of the maze. In the “No-maze condition”, participants engaged in a task that was formally identical to the T-maze task except that the imperative and feedback stimuli were displayed on a scrambled screen. The task consisted of 6 blocks of trials (50 trials per block) alternating between T-maze and No-maze conditions, the order of which were counterbalanced across subjects *(please see SOM for behavioural performance of the Maze conditions).*

### Spatial Ability Measures

Following Baker and Holroyd (2013), individual differences in spatial ability were measured prior to the fMRI session using the complex-maze reconstruction task, a virtual environment test battery designed to assess core strategies used by humans to navigate^35^, and a questionnaire assessing individual difference in in daily orientation and navigation^26^. See supplementary online material (SOM) for full details. The complex-maze consisted of sets of T-junctions arranged such that each of eight feedback locations could be reached from the starting position by way of a sequence of 3 left and right turns. Subjects were required to reconstruct the maze from memory after an allocated number of trials and then classified into two groups: “Non-drawers” (scores > = 1: participants who could not reconstruct from memory) and “Drawers” (scores = 0: those that could) (SOM).

### Region of interest analysis: (please see SOM for fMRI acquisition parameters)

We analysed fMRI data using SPM8 (www.fil.ion.ac.uk/spm). Functional images were spatially aligned to the first image in the series and then co-registered with the T1 image. Images were normalized to the Montreal Neurological Institute (MNI) template and smoothed using a Gausian kernel of 8 mm full width half-maximum. The design matrix convolved the experimental design with a hemodynamic response function. The model was estimated using proportional scaling over the session to remove global artifacts. Each single event was modeled as a hemodynamic response function (HRF) and its temporal derivative. High-pass Filter of 128 s were applied to the timeseries data. For each individual, t-contrasts were computed across T-maze and No-maze conditions, and across the T-maze right alley and T-maze left alley conditions. Thereafter we conducted whole brain, second-level, random-effects analyses on t-contrasts from the individual data. Activated regions were labeled based on MNI coordinates using xjview. ROI analyses were conducted to obtain activation values (the resultant parameter estimates for the contrast) from individual data for the right PHC using MarsBaR^36^. ROIs were defined by the Automated Anatomical Labeling (AAL) pickatlas measurement (WFU_Pickatlas)^37^. Follow-up analyses were calculated as the correlation between right PHC and individual measures on spatial ability. To test our hypotheses, a threshold of p < .05 family-wise error correction (FWE) was used in all significance tests. For exploratory purposes we also examined the data with an uncorrected threshold.

## Author Contributions

T.E.B. and A.U. carried out the functional neuroimaging data collection. T.E.B., A.U. and A.K. carried out neuroimaging data processing and analysis. T.E.B., C.B.H. and A.K. designed the study. T.E.B., C.B.H. and A.U. prepared the manuscript.

## Supplementary Material

Supplementary Information Supplementary Online Material

## Figures and Tables

**Table 1:** 

	Cluster Level	Peak Level	Size	Location (x y z)	Anatomical Label
	*p-value (FWE-corr)*		*(Voxels)*	*(Peak MNI coordinate)*	*(Peak MNI coordinate Region)*
Maze	<.001	t = 15.04, p <.001	**111**	34, −74, 22	**Right Middle Temporal Cortex**
			*67*		*Sub-Gyral*
			*10*		*Superior Occipital Cortex*
			*4*		*Middle Occipital Cortex*
	<.001	t = 11.65, p <.001	**122**	16, −74, 46	**Right Precuneus**
			*5*		*Superior Parietal Lobule*
	<.001	t = 9.15, p <.01	**69**	−32, −84, 20	**Left Middle Temporal Cortex**
			*47*		*Middle Occipital Cortex*
			*14*		*Sub-Gyral*
			*7*		*Superior Occipital Cortex*
			*2*		Cuneus
	<.001	t = 9.37, p <.01	**32**	−30, −46, −8	**Left Parahippocampal Cortex**
			*2*		*Left Fusiform*
	<.001	t = 8.82, p <.01	**20**	26, −40, −12	**Right Parahippocampal Cortex**
No Maze	<.005	t = 9.58, p <.005	**28**	16, −94, 18	**Right Cuneus**
			*15*		*Middle Occipital Gyrus*
	<.005	t = 8.37, p <.01	**24**	−16 −96 −12	**Left Middle Occipital Cortex**
			13		*Cuneus*
