# Supplementary material for: Rightward-biased hemodynamic response of the parahippocampal system during virtual navigation
Source: Sci Rep. 2015 Mar 12;5:9063. doi: 10.1038/srep09063 (PMC4356951; doi:10.1038/srep09063)
Supplement: Supplementary Information — Supplementary Online Material [file srep09063-s1.doc]

Supplementary Online Material: Rightward-biased hemodynamic response of the parahippocampal system during virtual navigation

Travis E. Baker^1*^, Akina Umemoto^2^, Adam Krawitz^2^,

and Clay B. Holroyd^2^

^1^Department of Psychiatry

University of Montreal

Quebec, Canada

^2^Department of Psychology

University of Victoria

British Columbia, Canada

*Corresponding author:

postal address:

Centre de recherche du CHU Ste-Justine

Université de Montréal

3175 Chemin de la Côte Sainte-Catherine

Montreal, H3T 1C5

Email: travis.e.baker@gmail.ca

Telephone: 1-514-867-9676

**Supplementary Online Material**

**Methods**

*Reconstruction Complex T-maze Task*. The complex T-maze (CT-maze) was identical to the virtual CT-maze used in Baker and Holroyd (2013) except that EEGs were not recorded. In brief, The CT-maze consisted of sets of T-junctions arranged such that each of eight feedback locations could be reached from the starting position by way of a sequence of 3 left and right turns (Figure S1). As in Baker and Holroyd (2013), participants navigated the maze in a highly constrained fashion: Each left or right button press rotated the subject’s perspective 90° to the left or right, respectively, to view the next junction point; then another blue arrow appeared and remained on the screen until participants made their next choice. As shown in Figure 1, the texture of the walls alternated throughout the maze to provide visual cues of the different locations within the maze. The subject’s virtual position was always located along the midline of the corridor. This sequence of events occurred three times on each trial for a total of 8 possible paths (e.g., [right, right, right]; [right, right, left]; [right, left, right]; [right, left, right]; etc.). Immediately following the third response, an image of the left or right alley end appeared, followed by the onset of the feedback stimulus (apple or orange). Participants were told that the presentation of one type of fruit indicated that the alley they selected contained 5 CAN cents (reward), and that the presentation of the other type of fruit indicated that the alley they selected was empty (no reward). The experiment consisted of 4 blocks of 25 trials each separated by self-paced rest periods.


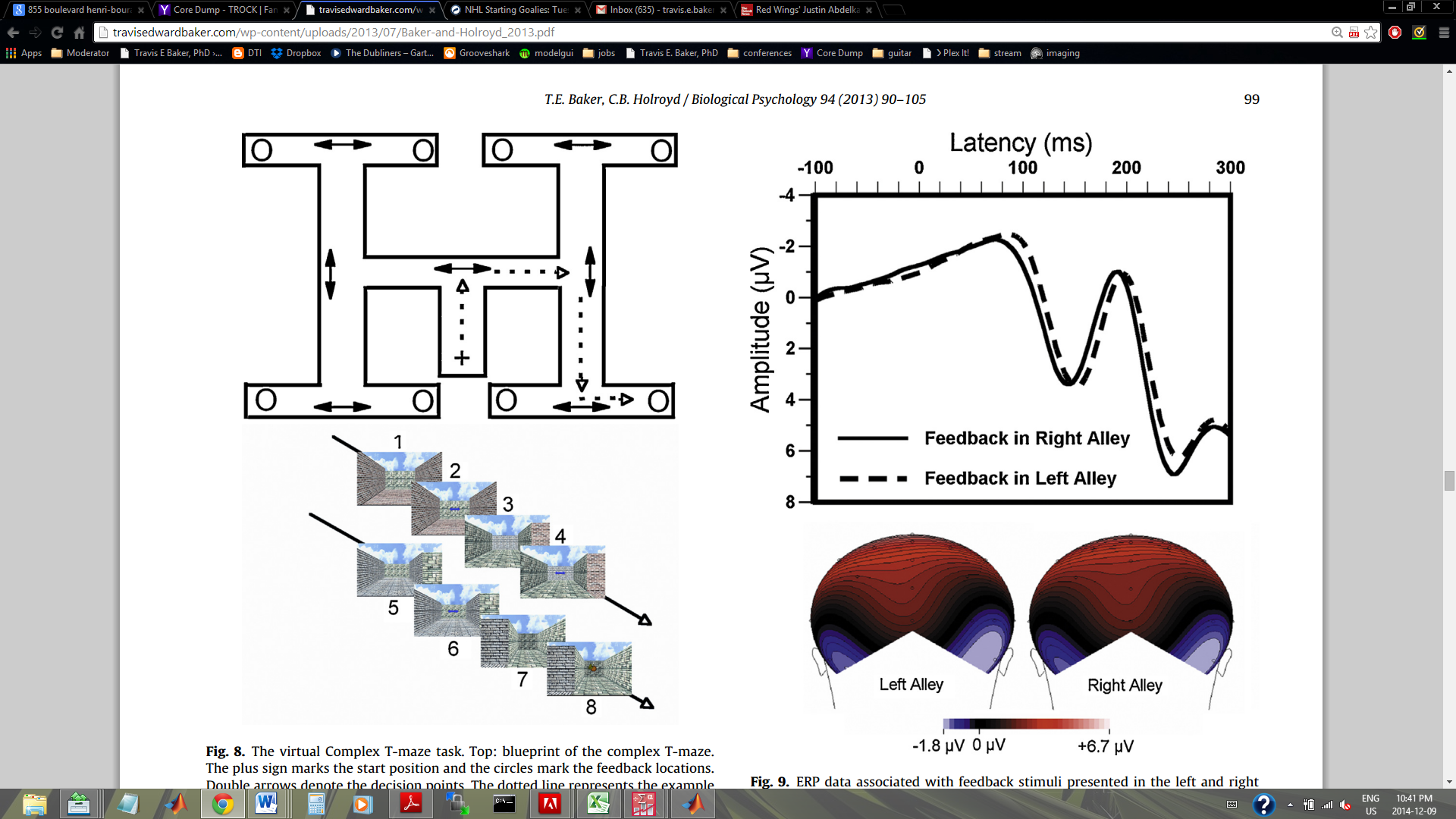


Figure S1. Adapted from Baker and Holroyd, 2013. Fig. 8. The virtual Complex T-maze task. Top: blueprint of the complex T-maze. The plus sign marks the start position and the circles mark the feedback locations. Double arrows denote the decision points. The dotted line represents the example path (right, right, left) shown in the bottom figure. Bottom: stimuli viewed from the participants’ perspective as they move through the complex T-maze on an example trial (1: Start image, 2: First decision arrow, 3: Alley image following right turn, 4: Second decision arrow, 5: Alley image following right turn, 6: Third decision arrow, 7: End alley image following left turn; 8: Feedback onset).

To examine individual differences in spatial memory, participants were asked to draw the spatial layout of the CT-maze from memory after each block of trials. They were not told of this requirement during the initial instructions and were not given a time limit to complete it. Reconstruction accuracy was determined by dividing the sketch into four quadrants and then scored as follows: 0 = no errors, 1 = at least one error was found in one quadrant, 2 = errors were found in two quadrants, 3 = errors were found in three quadrants, 4 = errors were found in four quadrants. These scores were used to classify participants into two groups: “Non-drawers” (scores >=1) and “Drawers” (scores = 0). Because subjects were not aware of the tasks requirement following the first block of trials, we used task performance following the second block of trials to categorize subjects as either Drawers, participants who reconstructed the maze perfectly, or Non-Drawers, participant who made more than 1 error on the second attempt to draw the maze.

*Santa Barbara Sense of Direction scale.* Following the CT-Maze task, participants were asked to complete a modified version of the Santa Barbara Sense of Direction scale (SBSOD)^1^, which consisted of 15 statements that a participant rates on a scale ranging from 1 (strongly agree) to 7 (strongly disagree)^2^. Prior to analysis, scores were transformed so that low scores indicated more perceived difficulties in daily orientation and navigation.

*Getting Lost Virtual Environment Test Battery.* Following the SBSOD, subjects were asked to perform a comprehensive battery administered through an internet website testing different orientation strategies in virtual environments ([www.gettinglost.ca](http://www.gettinglost.ca/); the “Getting Lost” Virtual Environment Test Battery: ^2 3^). Performance was recorded and stored in a password-protected database for offline analysis. The testing battery presented here was composed of tests assessing five of the core orientation skills used by humans that have been identified from neuropsychological and experimental literature: *Landmark Recognition, Heading Orientation, Sequence Matching, Path Integration, Cognitive Map Formation and Cognitive Map Use ^2 3^* . See Arnold et al. (2013) for information regarding the stimuli, procedure, and analysis methods used in each test, as well as the cognitive and neural processes that have been associated with each task.

*Image acquisition and preprocessing.* Functional images of the whole brain were acquired in an axial orientation using a 1.5-Tesla (General Electic) MRI scanner. Blood oxygenation level-dependent functional images were collected by using a T2* weighted gradient echo planar imaging (EPI) sequence (TR=3 s, TA=1.43 s, flip angle = 80 degrees), with an in-plane resolution of 3.75mm by 3.75 mm (64 X 64 matrix; 220 X 220 field of view). Twenty-three 5 mm slices (skip .6 mm between slices) were acquired inferior (bottom) to superior (top) in an interleaved order. In-plane structural scans were collected by using a T1-weighted sequence in the same orientation as the functional sequences to provide detailed anatomic images aligned to the functional scans. A high-resolution structural MRI sequence was also acquired for the purpose of normalization. Distortion in EPI images was corrected on the basis of estimated parameters of the phase map. After discarding the first 4 images, the next 216 successive images in each run were subjected to analysis.

The fMRI data were analyzed using standard procedures implemented in Statistical Parametric Mapping (SPM8: the Wellcome Department of Imaging Neuroscience, London, UK). First, the functional images were slice-timing corrected using Fourier phase-shift interpolation with the first slice as reference and then motion corrected and resliced using least-squares 6-parameter rigid-body transformation. The structural scan was then coregistered with the functional scans using affine transformation and resliced. In particular, EPI images were registered to each participant’s T1 in-plane images and then to the high resolution images. The high-resolution images were normalized to a standard template supplied with SPM, which are written to Montreal Neurological Institute (MNI) space with 12-parameter affine registration followed by a nonlinear deformation (25 iterations, nonlinear regularization = 10) with the structural scan as source image and SPM8’s MNI Avg152 T1 at 2 mm^3^ with associated weighting mask as template. Finally, the normalized images were smoothed with an 8-mm^3^ FWHM Gaussian kernel.

*Intraparticipant analysis*. The first-level analysis of the preprocessed fMRI data was performed using SPM8. A general linear model (GLM) was run for each subject with a canonical hemodynamic response function (HRF) and its temporal derivative. Low-pass (HRF) and high-pass (mean cutoff period of 128 s) frequency filters were applied to the timeseries data and restricted maximum likelihood (ReML) for model estimation. The model included a constant term, 6 motion regressors using the parameters of the motion correction performed during preprocessing, and 8 event-related regressors to model activation during the feedback period.

The feedback period for each trial was classified on whether the feedback was presented in the Maze, or No-maze, whether the feedback was presented following a right hand response or left hand response, and whether the feedback was a reward or no-reward cue. This provided eight regressors (Maze right reward/no reward, Maze left reward/no reward, No-maze right reward/no reward, No-maze left reward/no reward) plus a seventh regressor (NoResponse) for trials in which no response was made, regardless of the trial type. All events were aligned to the time of feedback onset. Contrasts of interest were defined for changes in brain activity during the feedback period. The Maze effect was defined as the difference in brain activation associated with feedback presented in the Maze condition versus feedback presented in the No-maze condition. For comparison of activations with a right response versus with a left response for the Maze condition, the Alley effect was defined as the difference in the Maze effect for feedback presented in the right alley compared to feedback presented in the left alley.

To determine which brain areas were more strongly activated in the Maze condition vs. No-maze condition, and Maze left turn vs. Maze right turn, the parameter estimates for each condition and for the difference between the conditions were calculated from the least mean square fit of the time series data to the model. Images of parameter estimates representing event-related activity at each voxel for each condition and each subject were created. Linear contrasts of coefficients for each participant were entered into a second level random-effects analysis by applying t-tests between the contrast images to create a group statistical parametric map (SPM). An SPM of voxels showing a significant response to stimulus presentation between the conditions were created. A False Discovery Rate (FDR)-corrected statistical threshold of p < 0.05 was used in all analysis to control for multiple comparisons. The results were overlaid on a 3D-rendered normalized brain in MNI space. We confirmed the location of visually identified BOLD clusters using automated anatomical labelling (MNI) atlas in SPM tool box: xjView (<http://www.alivelearn.net/xjview>).

For exploratory purposes, we also included a post hoc analysis to examine whether spatial ability factors (SBSOD, Getting Lost performance) would correlate with the BOLD signal of the significant clusters obtained from the Maze/No-maze contrast. In particular, the functional data associated with each cortical cluster identified from the Maze vs. No maze contrast was correlated across subjects with the general spatial navigation ability factors.

**Results**

*T-Maze and No-maze Behavioral Performance.* In regards to Maze and No-maze performance, there were no significant differences in reaction time between left responses and right responses in the Maze condition (Left = 366 ms; Right = 364 ms, p=.70) and the No-maze condition (Left = 372 ms; Right = 355 ms, p=.14), nor any significant differences in reaction time between Maze left vs. No-maze left (p=.56) and Maze right vs. No-maze right (p=.62) conditions. Further, there were no significant differences in number of choices between left responses and right responses in the Maze condition (Left =47; Right = 44, p=.30) and the No-maze condition (Left = 48; Right = 44 ms, p=.17), nor any significant differences in number of choices between Maze left vs. No-maze left (p=.54) and Maze right vs. No-maze right (p=.90) conditions.

*Santa Barbara Sense of Direction scale*. Prior to analysis, the SBSODS items were score and transformed so that low scores indicated a lack of perceived real-world orientation competence. Responses to the SBSODS questionnaire were included in a factor analysis to determine the factor structure for the purpose of calculating individual scores specific to difference aspects of perceived spatial ability. We calculated an exploratory factor analysis (EFA) using a Varimax rotation. Six factors were constrained, and were retained using the eigenvalues greater than 1 criterion. The eigenvalues for the first six factors accounted for 86.1% of the total variance. Defining salient loadings as those items showing a standardized factor loading of > .50 (i.e., “fair” or better), most of the SBSOD items loaded. Given the small number of participants in this study, the factor analysis should be considered as very exploratory and only one criterion used in selecting items for the final scale (standardized factor loading of > .50). Target specification for the assignment of SBSOD items to each respective factor are as follows: (a) factor 1 (Perceived Spatial Ability: 35.7 % of the variance) would include items 1, 4, 5, 11, 13, and 15 (Mean score = 23.1); (b) factor 2 (Object Recognition: 17.8 % of the variance) would include items 1, 4, and 8 (Mean score = 22.6); (c) factor 3 (Route Finding: 12.0 % of the variance) would include items 2, 8, 12, 24, and 14 (Mean score = 23.0); (d) factor 4 (Face Recognition: 7.6 % of the variance) would include items 16, 17, and 20 (Mean score = 14.8); (e) factor 5 (Map Reading: 4.6 % of the variance) would include items 7, and 9 (Mean score = 8.9); and (f) factor 6 (Orientating: 4.2 % of the variance) would include items 10, and 23 (Mean score = 8.6).

It is interesting to note that 7 items loaded on the first factor, Perceived Spatial Ability, and that Item 1 (‘‘My sense of direction is very good’’), and Item 2 (“I am very good at giving direction) had the highest loading on the first factor (>.90), comparable to results obtained by the original validation study ^1^. This was the main factor of interest, which we termed Perceived Spatial Ability, that we used in the subsequent analysis. Given the exploratory nature of the factor analysis, the other factors should be interpreted with caution.

A one-way analysis of variance (ANOVA) revealed that the Non-drawers, on average, perceived themselves as having more difficulties in spatial ability (M = 17.75, SE = + 3) than Drawers did (M = 28.5, SE = + 3), F(1, 14) = 5.88, p < .05, η2 = .30. When controlling for Sex, the difference between Drawers and Non-drawers was strengthened, F(1, 12) = 10.94, p < .005, η2 = .46, and Sex displayed a significant difference between Spatial Ability, such that Females, on average, reported more difficulties in daily orientation and navigation (M = 17.2, SE = + 2) than Males did (M = 29, SE = + 2), F(1, 16) = 12.07, p < .005, η2 = .50. However, the “Drawer by Sex” interaction was not significant, precluding any firm conclusions about the relationship between sex and spatial ability on this task. Further, no differences were found between Drawer groups on any other SBSOD factors.

*Getting Lost*: Scores on Landmark Recognition, Heading Orientation, Sequence Matching, Path Integration and Cognitive Map Use represent the number of correct responses across each test; thus higher scores indicate better performance. Conversely, scores on Cognitive Map Formation represent the number of trials until participants correctly located each of the four landmarks on an aerial map of the city, so lower scores indicate better performance. Data were lost for 3 subjects due to computer malfunctions. Test results were as follows: 1) Landmark Recognition (maximum trials = 10, n=13, M = 9.69, SE = 0.79); 2) Heading Orientation (maximum trials = 10, n=13, M = 9.23, SE = .34); 3) Sequence Matching (maximum trials = 10, n=16, M = 9.87, SE =.08), 4) Path Integration (maximum trials = 10, n=14, M = 7.2, SE = .54), 5) Cognitive Map Formation (maximum trials = 20, n=15, M = 9.33, SE = 1.41) and 6) Cognitive Map Use (maximum trials = 10, n=15, M = 5.5, SE = .38). Importantly, it should be noted that performance on Landmark Recognition, Heading Orientation and Sequence Matching was near ceiling. For this reason we excluded these test results from the subsequent ANOVAs. These ceiling effects were also observed in a previous study, and it has been suggested that future versions of the tests should ensure greater individual variability in performance ^2^.

To test whether Drawer type differed in performance on each of the tests, a multivariate ANOVA was run using Path Integration and Cognitive Map Formation performance as dependent variables with Sex included as a covariate. The results of the this analysis revealed that the Non-drawers, on average, displayed worse performance on the Path Integration Task (M = 6.0, SE = + .73) than Drawers did (M = 8.1, SE = + .64), F(1, 10) = 4.77, p < .05, η2 = .32. Further, Non-drawer performed worse on the Cognitive Map Formation Task (M = 12.66, SE = + 1.83) compared to the Drawers (M = 6.2, SE = + 1.6), F(1, 11) = 8.2, p < .01, η2 = .39. No sex differences were observed on any of the tasks, comparable to the results obtained by Arnold and colleagues (2013).

References

1. Hegarty,M., Richardson,A.E., Montello,D.R., Lovelace,K., & Subbiah,I. Development of a self-report measure of environmental spatial ability. *Intelligence* **30**, 425-447 (2002).
2. Arnold, A.E.G.F., Burles, F., Krivoruchko, T., Liu, I., Rey, C.D., Levy, R.M. & Iaria, G. Cognitive mapping in humans and its relationship to other orientation skills. *Experimental Brain Research*, **224**, 359-372 (2013).
3. Iaria,G., Petrides,M., Dagher,A., Pike,B., & Bohbot,V.D. Cognitive strategies dependent on the hippocampus and caudate nucleus in human navigation: variability and change with practice. *J. Neurosci*. **23**, 5945-5952 (2003).
